# Digitalization of Enzyme-Linked Immunosorbent Assay with Graphene Field-Effect Transistors (G-ELISA) for Portable Ferritin Determination

**DOI:** 10.3390/bios14080394

**Published:** 2024-08-16

**Authors:** Melody L. Candia, Esteban Piccinini, Omar Azzaroni, Waldemar A. Marmisollé

**Affiliations:** Instituto de Investigaciones Fisicoquímicas Teóricas y Aplicadas (INIFTA), Departamento de Química, Facultad de Ciencias Exactas, Universidad Nacional de La Plata (UNLP), CONICET. 64 and 113, La Plata B1900, Argentina; melodycandia@inifta.unlp.edu.ar (M.L.C.); estebanpiccinini@inifta.unlp.edu.ar (E.P.)

**Keywords:** graphene field-effect transistor, bioelectronic immunosensing, G-ELISA, ferritin

## Abstract

Herein, we present a novel approach to quantify ferritin based on the integration of an Enzyme-Linked Immunosorbent Assay (ELISA) protocol on a Graphene Field-Effect Transistor (gFET) for bioelectronic immunosensing. The G-ELISA strategy takes advantage of the gFET inherent capability of detecting pH changes for the amplification of ferritin detection using urease as a reporter enzyme, which catalyzes the hydrolysis of urea generating a local pH increment. A portable field-effect transistor reader and electrolyte-gated gFET arrangement are employed, enabling their operation in aqueous conditions at low potentials, which is crucial for effective biological sample detection. The graphene surface is functionalized with monoclonal anti-ferritin antibodies, along with an antifouling agent, to enhance the assay specificity and sensitivity. Markedly, G-ELISA exhibits outstanding sensing performance, reaching a lower limit of detection (LOD) and higher sensitivity in ferritin quantification than unamplified gFETs. Additionally, they offer rapid detection, capable of measuring ferritin concentrations in approximately 50 min. Because of the capacity of transistor miniaturization, our innovative G-ELISA approach holds promise for the portable bioelectronic detection of multiple biomarkers using a small amount of the sample, which would be a great advancement in point–of–care testing.

## 1. Introduction

Ferritin is a protein, first isolated in 1937 and revealed years later to be composed of protein, nucleic acids and more than 20% iron [1,2,3]. Furthermore, ferritin has 24 subunits divided into ferritin heavy chain (FTH) and ferritin light chain (FTL) subunits with an iron core [2,3,4,5]. The main function of ferritin is the regulation of iron in tissues, where it is stored as a cytosolic protein [3,4,6,7]. Likewise, the relationship of serum ferritin with iron concentration was exposed in a study, where it was confirmed that serum ferritin decreased in patients with iron deficiency and increased in patients with iron overload, which is evidence that the concentration of iron regulates the level of serum ferritin [3,8]. Also, elevated serum ferritin levels can be an indicator of various diseases, such as hepatitis, cancer, cirrhosis, leukemia, rheumatic and inflammatory diseases [3,9,10,11,12,13]. Furthermore, ferritin has recently been proposed as a severity marker in the diagnosis of COVID-19 [14,15].

The quantification and purification of human serum ferritin using an immunoassay was demonstrated in 1972 [16]. After that, several methods were developed, such as radioimmunoassay, enzyme-linked immunosorbent assays (ELISA), electrochemiluminescence, mass spectrometry and surface plasmon resonance [17,18,19,20]. However, these methods require sophisticated and expensive instruments, as well as trained personnel, and are hardly miniaturized to achieve multiplex and portable formats [20,21,22]. Bioelectronic sensors have emerged as a potential solution to these issues. Among the different types of bioelectronic sensors, those based on field effect transistors (FETs) have shown advantages for the detection of molecules with high sensitivity and low concentrations of analytes [23,24,25]. In addition, FETs have a high potential for the development of biosensing devices because their electronic measurements can be easily digitized and thus facilitate patient monitoring [26,27]. In particular, graphene-based FETs (gFETs) are promising for bioelectronic transducers since graphene is a two-dimensional carbon-based semiconductor material with good chemical stability, high conductivity, high carrier mobility and a large specific area [28,29,30]. In recent years, these devices have gained relevance since they have made it possible to detect ions and charged biomolecules with a high degree of sensitivity in real time and without resorting to the use of markers [31,32,33]. Their operating principle is based on the use of graphene as a semiconductor channel between the drain and source electrodes. Likewise, the conductivity of the semiconductor channel (graphene) between the drain and source contacts can be modulated by applying a potential difference between the gate and source electrodes (V_G_). The application of the potential V_G_ produces a change in the concentration of charge carriers of the graphene and this affects the current between drain and source contacts (I_DS_). When the conductivity is minimal (Dirac point or inversion potential, V_I_), the valence band is completely filled and the conductance band is completely unoccupied [34,35].

There are several ways to synthesize graphene for transistor fabrication, for example using chemical vapor deposition (CVD) and reduced graphene oxide (rGO), and each one results in devices with different inherent properties [36]. If monitoring pH change or enzymatic reaction is desired, the use of gFETs fabricated from rGO [37,38] will present sensitivity advantages over those fabricated with CVD [39]. This behavior is observed because reduced graphene oxide (rGO) exhibits remaining functional groups on the surface of graphene, such as -OH and -COOH, that could generate a change in the surface charge of graphene, and the change in the diffuse double layer, with the variation of the pH that can lead to gating effects [37,40,41,42,43]. These properties can be used for the development of biosensors. For biosensors that use high-affinity recognition elements, such as antibodies and aptamers, it is also necessary to consider the chemical approach to attaching the recognition elements to the graphene surface [23,44,45,46,47]. These recognition elements must retain their activity after immobilization and must allow accessibility to their active sites. In this regard, the modification by covalent bonding could generate defects in the sp^2^ chemical structure of graphene, hindering the charge transport and thus affecting the sensor response [48,49,50]. An alternative is the assembly by π–π interactions with graphene, as occurs with monopyrenes, although they are susceptible to desorption, mainly if hydrophilic or highly-charged molecules are attached. In regard to this, our work group has recently described an assembly for the immobilization of recognition and antifouling elements in graphene using a multivalent heterobifunctional approach based on vinylsulfonated polyethyleneimine (VS-PEI). In this strategy, recognition elements and polyethylene glycol (PEG) are covalently attached to VS-PEI and all the assembly is anchored to graphene through multiple π-π interactions [51,52]. It was demonstrated that VS-PEI was more stable and has better antifouling properties than the one based on monopyrene. In addition, this new strategy presents a covalent-like stability even after being exposed to strong surfactant solutions [51,52].

Although gFETs present a set of attractive characteristics for label-free biomarker immunodetection, this strategy is compromised when the target is present at low concentrations, or concentrations below the affinity constant K_D_ of the recognition element. This is because the transduction mechanism of label-free gFETs is based on the modulation of the electric field in the vicinity of graphene when a target biomolecule is recognized. As previously demonstrated, the response of the gFET is proportional to the mass of the recognized target, making its limit of detection near to 2 ng/cm^2^ [52]. To overcome these challenges, amplified detection methods must be developed. An attractive alternative is coupling enzyme-linked immunosorbent assay with pH responsive transistors using a reporter enzyme that catalyzes a pH-changing reaction [44,53]. This approach exhibits two outstanding features: detection enhanced by an enzymatic amplification and straightforward digitalization of the ELISA readout. Although the use of ELISA-like amplification coupled with indium oxide (In_2_O_3_) transistors was previously demonstrated, these devices usually have lower conductivity and transconductance than graphene FET, making the development and fabrication of miniaturized and portable readout devices more costly. On the other hand, nowadays, there are companies with commercially available portable readout stations for graphene FET sensors with very simple usability [52,54,55,56,57]. In this work, the amplified and portable detection of human ferritin was demonstrated through a sandwich ELISA protocol on a gFET device (G-ELISA). The graphene surface was modified with anti-ferritin antibodies using a multivalent heterobifunctional approach. Furthermore, urease was used as an ELISA reporter enzyme, which, in the presence of urea, catalyzes its hydrolysis generating an increase in pH close to the graphene surface. Thus, the gFET inherent capability of detecting pH changes transduce the presence of ferritin, which is easily digitalized. Moreover, the sensing performance of the G-ELISA was compared with those obtained for an unamplified gFET for ferritin detection.

## 2. Materials and Methods

### 2.1. Chemicals

Dimethylformamide (DMF), Na_2_CO_3_, HEPES, and KCl were purchased from Anedra (Los Troncos del Talar, Argentina). Divinylsulfone (DVS), 1-pyrenebuthanoic acid succinimidyl ester (PBSE), Polyethilenimine (PEI), PEG-NH_2_ (10 kDa) and human ferritin (CAS: 9007-73-2, catalog number: 341482, purity > 95% by SDS-PAGE) were purchased from Sigma-Aldrich (Darmstadt, Germany); sodium borate (from Parafarm), urea and NaCl (Biopack—Buenos Aires, Argentina), (KH_2_PO_4_ (Carlo Erba—Cornaredo, Italy), ethanolamine (Mallinckrodt—St. Louis, MO, USA) and anti-human ferritin heavy chain 1 (FTH1) monoclonal antibody (source: mouse IgG1, catalog number: 13,217-MM06) were purchased from Sino Biological (Houston, TX, USA). Commercial phosphate buffered saline 1× (PBS), Bovine Serum Albumin (BSA, 99%) and biotinamidohexanoic acid N-hidroxtsuccinimide ester (98%) were purchased from Sigma-Aldrich (Darmstadt, Germany), lyophilized streptavidin (15.28 U/mg) and urease (386 U/mg) were purchased from Serva (Heidelberg, Germany) and Calzyme Laboratories (San Luis Obispo, CA, USA) respectively.

### 2.2. Buffers

The following buffer solutions were used for different steps and procedures. PBS: 1× commercial phosphate buffered saline (pH 7.4); BBS: borate buffered saline (10 mM sodium borate, 140 mM NaCl, pH 9); M buffer: measurement buffer (0.1 mM HEPES 10 mM KCl, pH 6); W buffer: washing buffer (1× PBS + 0.05% Tween 20); B2 buffer: blocking buffer (100 mM Hepes + 0.05% Tween 20 + 0.1% BSA); and PBST-BSA buffer (1× PBS + 0.05% Tween 20 + 0.5% BSA).

### 2.3. Protein Solutions

Streptavidin (0.1 mg/mL), biotinylated anti-human ferritin monoclonal antibody (0.1 mg/mL, b-mAb-Ferritin), and the biotinylated urease (5 to 360 nM, b-urease) stock solutions were prepared in PBS. The urea (100 mM) stock solution was prepared in the M buffer. Ferritin solutions (0.05 to 10 nM) for ELISA-gFETs measurements were prepared in buffer PBST-BSA. Finally, the BSA solution (0.1%) was prepared in milliQ H_2_O as a blocking solution (B1 solution).

### 2.4. Measurement Set-Up

Commercial gFETs comprised chips containing reduced graphene oxide deposited on interdigitated gold microelectrodes as source and drain electrodes, with an Ag/AgCl coplanar gate microelectrode which was supplied by GISENS BIOTECH (Berkeley, CA, USA) [54]. Electrical characterization and FET performance of these gFETs was reported elsewhere [42,52,58]. Electrical measurements were performed using a Zaphyrus-W10 portable FET measurement station (GISENS BIOTECH), which has a detection chamber (500 µL volume cell) to confine the sensing solution to the chamber area of the gFET. Incubation and washing solutions were changed manually for all measurements. For gFETs modified with streptavidin or antibodies, measurements were performed by recording the drain–source current (I_DS_) as a function of time using V_GS_ set to −250 mV and V_DS_ set to 50 mV.

### 2.5. Measurement of Sensitivity to pH Changes

After manually mounting the gFETs in the Zaphyrus-W10 portable FET measurement station (Gisens Biotech, Buenos Aires, Argentina), solutions of different pH values ranging from pH 3 to 11 were introduced in the detection chamber. Then, I_DS_ vs. V_G_ curves were recorded using V_DS_ set to 50 mV.

### 2.6. Modification of the gFETs with Streptavidin

Commercial gFETs were incubated for 2 h in a 5 mM solution of 1-pyrenebutyric acid N-hydroxysuccinimide ester (PBSE) in dimethylformamide (DMF). Subsequently, they were washed with DMF and dried with N_2_. Then they were incubated for 1.5 h in 0.1 mg/mL streptavidin in PBS. Next, they were incubated for 2 h in 0.2 mM polyethylene glycol-amino (PEG-NH_2_) in PBS. Finally, they were incubated for 15 min in 100 mM ethanolamine (ETA) in PBS, washed with PBS and stored with a drop of PBS in the refrigerator (4 °C).

### 2.7. Urea Hydrolysis Measurements on b-Urease-Streptavidin gFETs

gFETs modified with streptavidin (Strept-gFET) were incubated with 30 µL of biotinylated urease (b-urease) at different concentrations for 13 min. They were then washed once with 300 µL PBS and four times with 300 µL of the measurement buffer (M buffer). Finally, 300 µL of M buffer was added and the I_DS_ vs. time was recorded. After four minutes, 3 µL of 100 mM urea was added. The measurement was continued for another eight minutes. Different concentrations of b-urease between 5 and 360 nM were used in these measurements.

### 2.8. Modification of the gFETs with mAb-Ferritin

Commercial gFETs were incubated for 2 h in a 5 mM PBSE solution in DMF. Subsequently, they were washed with DMF and dried with N_2_. Then, they were incubated for 1 h in 2 mg/mL polyethyleneimine (PEI) pH 10 aqueous solution, washed with milliQ water and dried with N_2_. Then, PEI-modified gFETs were incubated for 1 h in 5% divinylsulfone (DVS) in 0.5 M Na_2_CO_3_ buffer pH 11. Then, they were washed with water and dried with N_2_. Afterwards, the VS-PEI modified sensors were incubated for 4 h in the 0.1 mg/mL anti-human ferritin monoclonal antibody (mAb-Ferritin) in BBS pH 9. Next, they were incubated for 2 h in 0.2 mM PEG-NH_2_ in BBS pH 9. Afterwards, they were incubated for 15 min in 100 mM ETA in BBS pH 9. Finally, the gFETs modified with mAb-Ferritin (mAb-Ferritin-gFETs) were washed with PBS and stored in PBS in the refrigerator (4 °C).

## 3. Results and Discussion

### 3.1. pH Monitoring by gFET Readout

**Figure 1:**
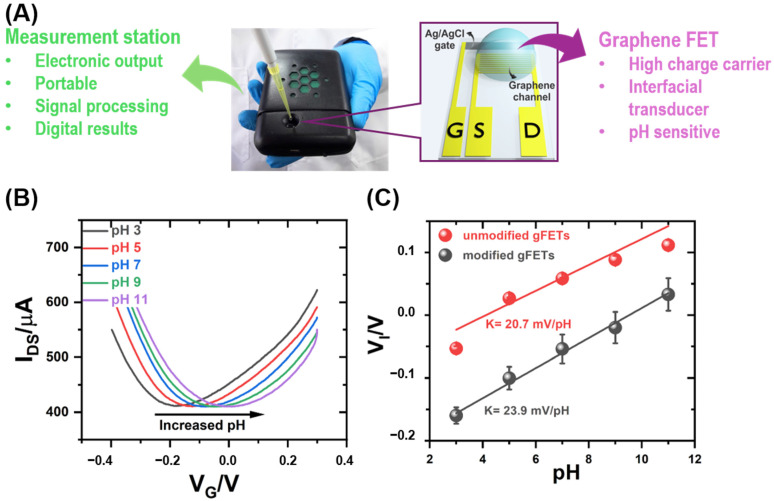
(**A**) Photograph of the portable measurement station and schematic of a gFET sensor. (**B**) Characteristic transfer curves for a streptavidin-modified gFET by varying the pH from 3 to 11 obtained at a V_DS_ = 0.05 V in a solution of 140 mM NaCl and 1 mM KH_2_PO_4_. (**C**) Change in Dirac potential as a function of pH for an unmodified gFET (red line) and modified gFET (black line). Slopes’ values from the linear fitting are reported for each gFET.

One of the objectives of this work was to incorporate a method similar to an ELISA assay to the transistor platform, and in this way amplify the recognition signal through enzymatic processes that lead to local pH changes. First, the sensitivity of gFETs to pH changes was studied. Transfer characteristic curves (I_DS_ vs. V_G_) were recorded in solutions of different pH values from 3 to 11 (Figure 1B). The application of an external electrical potential (V_G_) changes the density of the charge carriers in the graphene channel. Thus, the variation of V_G_ promotes changes in the current I_DS_. Particularly, in the case of gFETs, a parabolic shape with a minimum is observed for the dependence of I_DS_ on V_G_. The V_G_ value at the minimum is referred to as Dirac or inversion potential (V_I_). At V_I_ there is a change in the nature of the charge carriers from holes (for V_G_ < V_I_) to electrons (for V_G_ > V_I_) [42]. As shown in Figure 1B, there is a shift in V_I_ to higher V_G_ values as the pH of the solution increases. V_I_ showed a linear dependence on pH, with a slope of 20.7 mV/pH for bare gFETs (Figure 1C and Figure S1). This result is in agreement with previous rGO FETs and is suitable for sensoring platforms based on local pH changes [34,37,59]. The pH–sensitivity of bare gFETs has been ascribed to the acid–base equilibrium of oxygen chemical functionalities remaining in graphene or present in the glass substrate, which induces changes in the electrostatic surrounding of the graphene-conducting channel. However, this pH dependence of the gFETs properties could be altered when modifying graphene with biorecognition elements. For this reason, the pH dependence of the gFET response after protein functionalization was also studied (Figure 1B,C).

Streptavidin was chemically anchored to the gFET surface as this protein would be used as a linker for the specific integration of biotinylated urease. The pH–dependence of the gFET modified with streptavidin (Strept-gFET) was then studied (Figure 1B). As in the case of bare gFETs, the characteristic curves of Strept-gFET also shifted to higher V_G_ values as the solution pH increased with a linear dependence of the V_I_ values on pH (see Supporting Information). The functionalization of the graphene surface did not only preserve the conducting properties of gFETs but it even increased the pH-sensitivity, as the slope in Figure 1C changed from 20.7 to 23.9 mV/pH unit. This effect has been extensively observed for polyelectrolyte and protein functionalization and it is ascribed to the presence of additional acid-basic chemical groups near the graphene whose electrostatic charge depends on the solution pH [42,60,61]. Thus, the enhanced pH sensitivity of protein-modified gFETs supports the idea of developing ELISA-like gFET immunosensors whose readout is based on the local pH changes caused by the urea hydrolysis mediated by the urease enzyme.

### 3.2. Study and Optimization of the Enzymatic and Indirect Readout

As previously reported, in the presence of urea, urease-functionalized gFETs displayed changes in the Dirac potential due to the local pH increment produced by the catalyzed urea hydrolysis [38,42]:

Urea + 3H_2_O → 2NH_4_^+^ + HCO_3_^−^ + OH^−^ (1)

**Figure 2:**
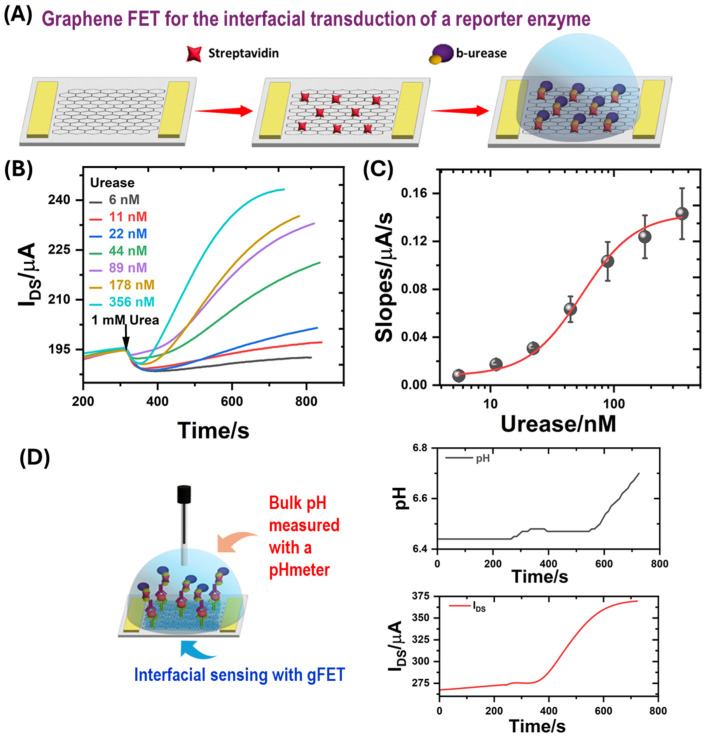
(**A**) Schematic of the Strept-gFET fabrication process. (**B**) Changes in I_DS_ after the addition of 1 mM urea for a Strept-gFET incubated with different concentrations of b-urease (V_DS_ = 50 mV, V_GS_ = −250 mV, 0.1 mM HEPES buffer with 10 mM KCl, pH 6). (**C**) Correlation between the slopes of the I_DS_ vs. time curves and the concentration of b-urease used in the modification of Strept-gFET. Bars correspond to the deviation of the slopes of two Strept-gFETs. The red line is the Hill-like model fitting. (**D**) G-ELISA scheme with capillary pH meter and comparison of changes in I_DS_ for a G-ELISA after incubation of 1 nM ferritin (V_DS_ = 50 mV, V_GS_ = −250 mV, 0.1 mM HEPES buffer with 10 mM KCl, pH 6) and measured pH changes with a capillary pH meter after the addition of 1 mM urea.

Therefore, after confirming the sensitivity of gFETs to pH changes, the viability of the enzymatic readout for an ELISA-like gFET method was investigated. First, a simple affinity-based system was used to optimize the enzymatic readout. Due to the specific and high-affinity interaction between streptavidin and biotin units, these biomolecules were used as proof of concept. Strep-gFETs were incubated in biotinylated urease (b-urease) solutions (Figure 2A). Biotinylation of urease was carried out as described in the Supporting Information (SI). Then, the gFET response in the presence of 1 mM urea (the enzyme substrate) was studied in real time (Figure 2B). Different concentrations of b-urease were used to find the optimal concentration. A V_G_ value of −250 mV was chosen due to the high transconductance in this region, which would yield marked variations in the I_DS_ because of the changes of graphene conductivity as the solution pH increased (see Figure 1B). Under these conditions, a clear increase in I_DS_ was observed after adding urea (Figure 2B). This result was consistent with an increment in the pH near the surface due to catalyzed hydrolysis of urea by the anchored urease.

As shown in Figure 2B, after adding urea, there was a near linear increment of I_DS_ on time for about 300 s. Interestingly, the changes in I_DS_ depended on the concentration of b-urease in the incubation solution. Particularly, the I_DS_ increment rate, after adding one mM urea, increased as a function of b-urease. This was consistent with the integration of a higher amount of enzyme on the Strept-gFETs, which was not detached in the subsequent washing steps due to the high biotin-streptavidin affinity. The slopes of I_DS_ vs. time (in the linear region, i.e., 300 s) for each urease concentration were estimated. These are related with the urea hydrolysis rate, which, in the presence of a high substrate concentration, would be dependent on the amount of enzyme in the system. Furthermore, by computing these slopes as a function of the b-urease concentration in the incubation step, a Hill-like plot was obtained, which allowed the fitting of an operational constant for the b-urease binding on the Strept-gFETs (Figure 2C) being K_D_ = 127.39 ± 64.05 nM. A maximum slope of 0.22 ± 0.06 µA/s corresponding to urease saturation was obtained. This operational K_D_ was higher (lower affinity) than the dissociation constant for the streptavidin–biotin system in solution, log (K_D_/M) ≈ −14 [62,63,64], as could be expected due to the fact that, in the present case, streptavidin was attached to the graphene surface and biotin moieties were bound to urease. On the other hand, using BSA instead of streptavidin attached to the surface of the gFET previous to the b-urease incubation step yielded negligible change in the I_DS_ after urea addition which indicates the importance of the specific binding to the surface-attached streptavidin.

The nature of the working buffer was also optimized. For this purpose, the response of the enzymatic reaction of b-urease/Strept-gFETs in different measurement buffers was studied (see SI file, Figure S2). The buffer with the best response was selected considering the slopes of the dependence of the I_DS_ as a function of time after urea addition. From these results, a working buffer of 0.1 mM HEPES in 10 mM KCl pH 6, a b-urease concentration of 175 nM and a urea concentration of 1 mM were chosen as optimal conditions to continue with the study of the G-ELISA.

Simultaneous measurements of I_DS_ and the solution pH using a capillary pHmeter (PHOENIX instrument, Garbsen, Germany; model 547-3901) were carried out in the same chamber for a G-ELISA using b-urease to reveal (Figure 2D) after the addition of one mM urea. Markedly, the gFET response reached a plateau many times faster than the solution pH measured with the pHmeter. This result indicates that a faster steady-state of reagents and product concentrations was reached on the graphene surface compared to the bulk solution; and, therefore, for short time period measurements, the pH at the graphene surface was higher than in the bulk solution. This set of results not only describes the optimized readout conditions, but also demonstrates the improved transduction capacity offered by gFETs due to their interfacial sensing compared to bulk measurement techniques.

### 3.3. ELISA-gFET for Ferritin Determination

**Figure 3:**
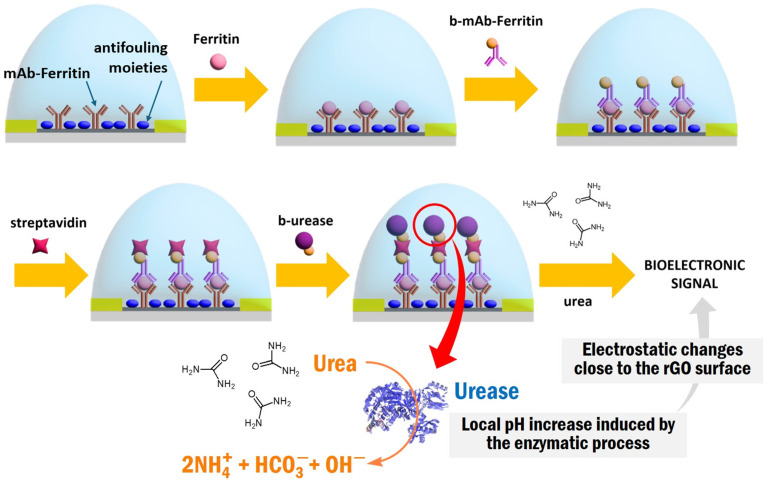
G-ELISA scheme for the detection of Ferritin.

After optimization of the urease concentration for the detection reaction, a G-ELISA configuration was developed for ferritin determination. In this strategy, monoclonal antibodies for ferritin (mAb-Ferritin) were employed as both primary and secondary antibodies. For this purpose, biotinylation of the secondary mAb-Ferritin was performed as described in the SI file. Within this strategy, the presence of the secondary biotinylated antibody was revealed by detecting the urease activity after successive incubation in streptavidin and b-urease solutions. Figure 3 shows a scheme of the whole gFET construction, which will be referred to as G-ELISA.

In the first step the primary mAb-Ferritin is anchored on the graphene surface by employing a heterobifunctional VS-PEI strategy as described above. The resulting mAb-Ferritin-gFETs are then exposed to the ferritin-containing samples and then a secondary biotinylated mAb-Ferritin (b-mAb-Ferritin) is used for the signal detection and amplification. After that, the modified gFET is incubated in 0.1 mg/mL streptavidin, and finally in 175 nM b-urease. As the secondary b-mAb-Ferritin would be anchored only if some ferritin molecules were previously bound to the device surface, the detection of urease activity would prove the presence of ferritin in the samples. Moreover, as the gFET response in the presence of urea depends on the amount of bound b-urease (as shown in the previous section), the G-ELISA construction would be able to quantify ferritin in the samples.

Several antifouling strategies were carried out to assure specificity. On the one hand, PEG-NH_2_ was used after the functionalization step with the primary mAb-Ferritin to integrate antifouling PEG units to the graphene surface (see Section 2.8). On the other hand, it was blocked with a BSA solution, while BSA and 0.05% Tween 20 were added to the blocking buffer and ferritin incubation buffer (Section S4). In this regard, the optimized procedure developed is summarized in Figure S3. In this strategy, mAb-Ferritin-gFETs mounted on the Zaphyrus-W10 are firstly blocked with 180 µL of B1 solution for 20 min. They are then washed three times with 300 µL of W buffer. A second blocking step is made with 30 µL of B2 buffer for 10 min followed by washing three times with 300 µL of W buffer. Then, they are washed five times with 300 µL of M buffer. Later, the measurement of the first control in the M buffer on time is started. After 4 min of measurement, 3 µL of 1 mM urea solution is added and another 10 min are measured (control 1, see SI file, Figure S3).

**Figure 4:**
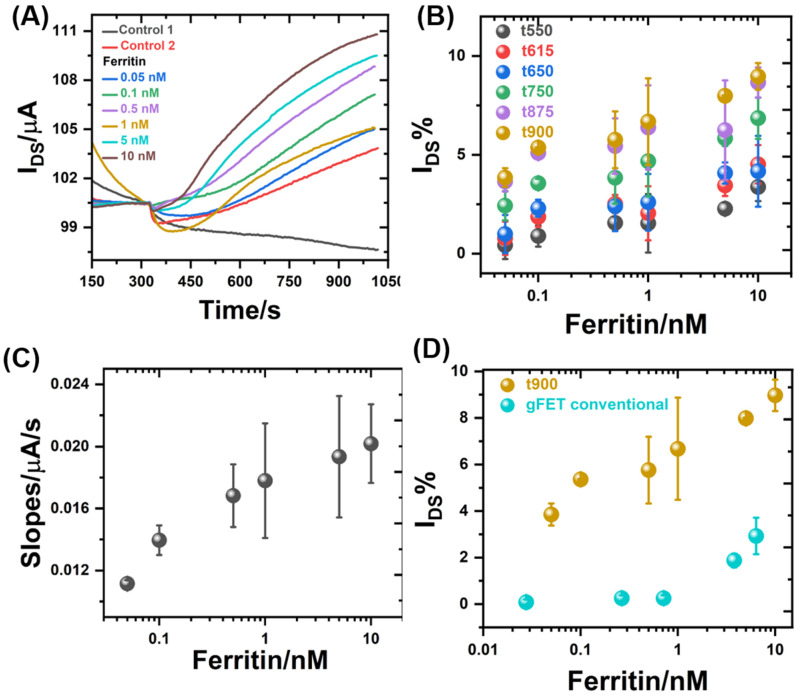
(**A**) Changes in I_DS_ for a G-ELISA after incubation with different concentrations of ferritin and the addition of 1 mM urea (V_DS_ = 50 mV, V_GS_ = −250 mV, 0.1 mM HEPES buffer with 10 mM KCl, pH 6). (**B**) I_DS_% as a function of the ferritin concentration calculated at different times, from 550 to 900 s. (**C**) Correlation between the I_DS_ slopes and the ferritin concentration obtained from the G-ELISA measurements and the detection with 1 mM urea. (**D**) Comparison of conventional I_DS_% gFET (V_DS_ = 50 mV, V_GS_ = −250 mV, HEPES buffer × 0.1 at pH 7.4) and I_DS_% at 900 s of G-ELISA as a function of ferritin concentration. Data for conventional gFET were reproduced from Piccinini et al., 2022 [52].

Then, the assay with different concentrations of the target (ferritin) is performed. For this stage the mAb-Ferritin-gFETs is incubated for 15 min in 30 µL of the ferritin solution in PBST-BSA, for 10 min in 30 µL b-mAb-Ferritin (secondary antibody), for 5 min in 0.1 mg/mL streptavidin, and finally for 5 min in 175 nM b-urease. After each incubation, the gFET is washed three times with 300 µL of W buffer. Finally, the measurement of the I_DS_ as a function of time is performed for the G-ELISA, where control 2 is performed following the incubation steps of the G-ELISA as shown in Figure 3, except for the incubation step with the solution ferritin. This procedure was successively repeated using different concentrations of ferritin from 0 to 10 nM. This ferritin concentration range covers both normal and pathological serum levels [65,66,67,68]. The detailed protocol is presented in the SI file (Section S5, Table S1). Curves of I_DS_ vs. time for each ferritin concentration are shown in Figure 4A. The gFET with the sandwich ELISA type of construction after all incubations (b-urease/streptavidin/b-mAb-Ferritin/Ferritin/mAb-Ferritin-gFET), is referred to as G-ELISA.

The I_DS_ of the G-ELISA increased when urea was added due to the increase in pH, induced by the enzymatic reaction of b-urease. Moreover, it can be seen that, as the ferritin concentration increased, the slopes of the curves of I_DS_ on time also increased. This plot shows that G-ELISA is able to detect the presence of ferritin in a dynamic concentration range (0.05 to 10 nM) encompassing normal ferritin levels, hyperferritinemia levels and severe ranges of cytokine storms, which are all clinically relevant [10,69].

As shown in Figure 4B, the relative change of I_DS_ in time well correlates with ferritin concentration, so this change could be used for determining the ferritin concentration. The I_DS_ changes with respect to the baseline (I_DS_ in urea aggregation) were obtained as follows [70]:

(2) $$I_{\mathrm{DSt}}\;\%=\frac{(I_{DSt}-I_{DS0})100}{I_{DS0}}$$

where I_DS_ is the current at the different times selected and I_DS0_ is the baseline current. The values of this parameter also depended on the time after the addition of urea. From Figure 4B, the values recorded after 900 s of urea addition show the higher response.

On the other hand, the slope of I_DS_ vs. time curves (indicative of the urea hydrolysis rate) also increased with the concentration of ferritin in the incubation solutions (Figure 4C). In principle, both parameters, the relative change in current (I_DS_%) and the slope of the current–time plots could be used as analytical parameters for sensing ferritin.

The limit of detection (LOD) of the G-ELISA was calculated from the I_DS_% at time 900 s and from the slopes. In both cases it was determined as three times the average of the standard deviation of four measurements of PBST-BSA (0 ferritin). The LOD calculated from the I_DS_% at 900 s was 0.01 nM, whereas the LOD calculated from the slope values was 0.05 nM. Both LOD values were significantly lower than those reached in previous studies in which detection was directly performed from the gFET response change after ferritin binding. That work reported a LOD of 0.53 nM and 0.23 nM for the functionalization of gFETs with antiferritin antibodies using the PBSE and PBSE/PEI/VS strategies, respectively [52]. In this regard, Figure 4D shows the comparison between the G-ELISA and the so-called conventional gFET (without an enzymatic amplified readout) for the different concentrations of ferritin. A clear I_DS_% increment can be observed for the G-ELISA method from 0.05 nM ferritin concentration, whereas the conventional gFET showed a response above 0.71 nM. According to these results, the amplification of the signal was clearly demonstrated when using an ELISA type methodology coupled with the gFETs. Stability studies show that the interfacial construction was relatively robust, which would even allow for the regeneration of the sensors (Figure S5 in Section S7).

The measurement configuration and features of G-ELISA were compared with previously ELISA-reported methods made using other type of FETs (Table 1). For instance, the ELISA strategy was coupled with indium oxide (In_2_O_3_) FETs for the detection of the biomarkers’ human immunodeficiency virus (HIV) p24 protein [44], interleukin-2 (IL-2) [45] and cardiac troponin I [46]. Transistor sensors prepared using In_2_O_3_ as semiconducting channel material have many times lower conductivity and transconductance than graphene FETs. Therefore, the I_DS_ recorded during the enzymatic readout was lower for In_2_O_3_ than for graphene devices. For example, Stern et al. reported an I_DS_-specific response of 68 nA for the detection of IL-2 with a In_2_O_3_ FET, while we obtained here up to 12 µA of specific response for the detection of ferritin with graphene FETs. In addition, for graphene FETs, I_DS_ is proportional to pH. On the other hand, for In_2_O_3_-FETs, Log (I_DS_) is proportional to pH, a relationship less appropriate for analytical applications. These differences have the result that the development and fabrication of miniaturized and portable readout devices for graphene FETs present a better cost-efficiency than those for FETs made of In_2_O_3_. For instance, bioelectronic In₂O₃ FET measurements are often recorded using standard semiconductor testing equipment commonly found in research laboratories, with a sale price of $20,000–80,000 USD. On the other hand, portable G-FET reading devices can be bought for less than 5% of the price of traditional In_2_O_3_ FETs measurement equipment. Indeed, nowadays, there are companies with commercially available portable readout stations for graphene FET sensors [52,54,55].

To evaluate economic viability, it is necessary to compare the cost of G-FET reader devices (less than $4000 USD) with other equipment currently used for ferritin quantification. A widely used and relatively low-cost reference method is ELISA determination using a multiwell spectrophotometer. These devices are sold at prices ranging from $20,000 to $50,000 USD. If we continue comparing the G-ELISA and the conventional ferritin ELISA kit, it can be seen that there is not only a large difference in the cost of the measurement devices but also a significant decrease in measurement time. A conventional ferritin ELISA takes between 4 and 5 h to fully develop, while the G-ELISA requires 1.3 h. Furthermore, commercial ferritin ELISA kits have a sensitivity of 1.33 nM (Sigma-Aldrich, Darmstadt, Germany; product number: RAB0197), while our system has a sensitivity of 0.01 nM. It is also worth noting that the ferritin ELISA kit costs approximately $700, while the gFET biosensor costs less than $15.

Additionally, ferritin quantification has also been reported using devices such as surface plasmon resonance (SPR) or bio-layer interferometry (BLI), which are significantly more expensive, costing between $80,000 and $250,000 USD. In this context, G-ELISA is a cost-effective methodology that could have high profitability margins.

An interference study was performed using concanavalin A (ConA) and glucose oxidase (GOx) instead of ferritin, both at a concentration of 10 nM (Figure S4). It can be seen that, despite having a response for both interfering agents, they did not exceed the response obtained by ferritin. This nonspecific response may also be due to the nonspecific binding of biotinylated urease, suggesting that further optimization could be conducted for blocking and washing the buffer composition (see Section S8, Figure S6).

Therefore, G-ELISA shows a high sensitivity, a low LOD and a wide detection range with a portable format setup and an easily digitalized readout (see video in Section S9). Furthermore, transistors show the remarkable possibility of making measurements in less than one hour. These advantages make G-ELISA an attractive method, not only for biochemist laboratories but also for point–of–care detection and quantification of protein targets.

## 4. Conclusions

In this study, we have introduced a novel functionalization method for graphene field-effect transistors (gFETs) to develop biosensors capable of quantifying ferritin. This involved immobilizing anti-ferritin antibodies on the graphene surface along with an antifouling agent. In addition, the electrolyte-gated arrangement was used in the gFETs, which allowed for operating in aqueous conditions with very low potentials, being essential for the detection of biological samples. Also, G-ELISA showed a good detection performance against changes in pH, which is essential in the present case due to the enzymatic amplification based on the urea hydrolysis catalyzed by urease. G-ELISA showed great potential for ferritin detection due to its very low LOD and high sensitivity in synthetic samples. The G-ELISA system is extremely promising and has several advantages compared with a commercial ELISA kit. On the one hand, G-ELISA reduced the time required to obtain results from the 4 to 5 h needed for a conventional ELISA to 1 h and 20 min. Additionally, the concentration range of ferritin that can be studied with G-ELISA spans from 0.05 to 10 nM, whereas the commercial ELISA kit has a measurement range of 1.8 to 444 nM. Our chosen study range included the normal serum ferritin range in healthy individuals. However, further work is needed on this system to reduce nonspecific binding and to enhance responses in blood serum. Finally, gFET technology would not only enable the direct digitization of ELISA assays but also, through the integration of microfluidic systems, could allow the miniaturization of multiplex immunodetection systems on a scale that is difficult to achieve with optical readout. Furthermore, this innovative biosensing strategy could be adapted for the detection of various biomolecules relevant to the diagnosis of different diseases. Thus, taking advantage of transistor miniaturization, our work paves the way for the development of multiplex and portable G-ELISA with digital results.

## Figures and Tables

**Table 1:** Comparison of different FET sensors amplified by ELISA type approach.

FET Material	I_DS_ Range	I_DS_ Dependence with pH	Urea for Readout	Analyte	Portable	Reference
In_2_O_3_	0–1 µA	Log (I_DS_) α pH	100 mM	HIV-1 p24	No	[44]
In_2_O_3_	0–2 µA	Log (I_DS_) α pH	10^5^:1 (urea to urease)	IL-2	No	[45]
In_2_O_3_	0–2 µA	Log (I_DS_) α pH	10 mM	Troponin I	No	[46]
rGO	100–500 µA	I_DS_ α pH	1 mM	Ferritin	Yes	this work

## Data Availability

The data presented in this work are available upon request from the authors.

## Supplementary Materials

The following supporting information can be downloaded at: https://www.mdpi.com/article/10.3390/bios14080394/s1, Figure S1: Characteristic transfer curves for an unmodified gFET varying the pH from 3 to 11 obtained at a V_DS_ = 0.05 V in a solution of 140 mM NaCl and 1 mM KH_2_PO_4_; Figure S2: Changes in I_DS_ after addition of 1 mM urea for a Strept-gFET incubated with different buffers (V_DS_ = 50 mV, V_GS_ = −250 mV); Figure S3: (A) G-ELISA scheme with antifouling strategies and measurement of control 1. (B) Changes in I_DS_ for a G-ELISA after incubation with different concentrations of ferritin and the addition of 1 mM urea, without the BSA blocking step (V_DS_ = 50 mV, V_GS_ = −250 mV, 0.1 mM HEPES buffer with 10 mM KCl, pH 6). (C) Comparison of G-ELISA slopes for two biosensors, one with the BSA blocking step and one without BSA blocking. (D) Changes in I_DS_ for a G-ELISA after incubation with different concentrations of ferritin and the addition of 1 mM urea, where washing steps were performed with PBS buffer pH 7.4 (V_DS_ = 50 mV, V_GS_ = −250 mV, 0.1 mM HEPES buffer with 10 mM KCl, pH 6). (E) Changes in the I_DS_ for a G-ELISA after incubation with different concentrations of ferritin and the addition of 1 mM urea, where washing steps were performed with PBST pH 7.4 buffer (V_DS_ = 50 mV, V_GS_ = −250 mV, 0.1 mM HEPES buffer with 10 mM KCl, pH 6). (F) Comparison of the slopes of G-ELISA for two biosensors, with different washing buffers, one with PBS pH 7.4 and the other with PBST pH 7.4; Figure S4: I_DS_% at 900 s of G-ELISA with a concentration of 10 nM of ferritin, ConA and Gox; Figure S5: (A) Changes in I_DS_ for a G-ELISA after incubation with different concentrations of ferritin and the addition of 1 mM urea (V_DS_ = 50 mV, V_GS_ = −250 mV, 0.1 mM HEPES buffer with 10 mM KCl, pH 6). (B) Changes in I_DS_ for a G-ELISA after incubation with different concentrations of ferritin and the addition of 1 mM urea (V_DS_ = 50 mV, V_GS_ = −250 mV, 0.1 mM HEPES buffer with 10 mM KCl, pH 6) for one biosensor recovered and measured 12 days later. (C) Comparison of G-ELISA slopes with 5 nM ferritin for two biosensors recovered and measured 12 days later; Figure S6: (A) Changes in I_DS_ for a mAb-Ferritin-gFET after incubation with b-urease, incubation with streptavidin and b-urease, and finally incubation with b-mAb-Ferritin, streptavidin, and b-urease and addition of 1 mM urea (V_DS_ = 50 mV, V_GS_ = −250 mV, 0.1 mM HEPES buffer with 10 mM KCl, pH 6). (B) Comparison of I_DS_% at 900 s of control 2 versus nonspecific binding of b-urease, streptavidin-b-urease, and b-mAb-ferritin-streptavidin-b-urease; Table S1: Detailed protocol for G-ELISA measurements.
