# Supplementary material for: Digitalization of Enzyme-Linked Immunosorbent Assay with Graphene Field-Effect Transistors (G-ELISA) for Portable Ferritin Determination
Source: Biosensors (Basel). 2024 Aug 16;14(8):394. doi: 10.3390/bios14080394 (PMC11352759; doi:10.3390/bios14080394)
Supplement: Supplementary file 1 [file biosensors-14-00394-s001.zip › biosensors-3072917-supplementary.pdf]

biosensors

Supplementary Material

Digitalization of Enzyme-Linked Immunosorbent Assay with Graphene Field-Effect Transistors (G-ELISA) for Portable Ferritin Determination

Melody L. Candia, Esteban Piccinini, Omar Azzaroni and Waldemar A. Marmisollé

S1. pH monitoring by reading unmodified gFETs

The sensitivity of unmodified gFETs to pH changes was studied. For this, the transfer characteristic curves (I_{DS} vs. V_G) were recorded in solutions with different pH values from 3 to 11. In Figure S1, a change in the minimum of the transfer curves at higher V_G values can be observed as the pH of the solution increases.

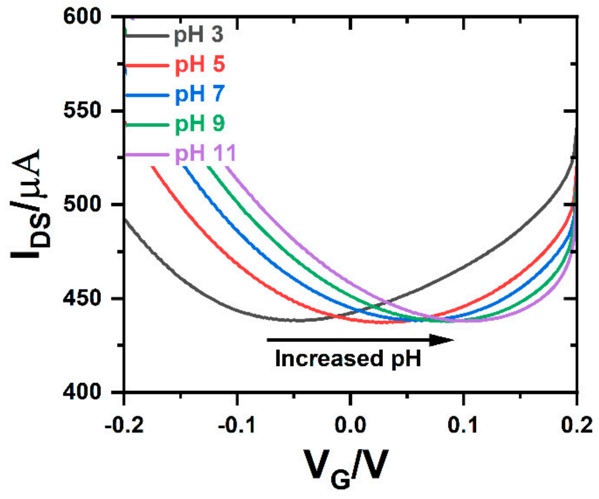

Figure S1. Characteristic transfer curves for an unmodified gFET varying the pH from 3 to 11 obtained at a V_{DS}=0.05 V in a solution of 140 mM NaCl and 1 mM KH_2PO_4.

S2. Protein Biotinylation

For urease biotinylation 1 ml of a 5 mg/ml urease solution in PBS (pH 7.4) and 0.1 ml of a 20 mg/ml NHS-LL-Biotin solution in DMF were prepared. A portion of 12 µl of the Biotinamidohexanoic acid N-hydroxy succinimide ester (NHS-LL-Biotin) solution was added to the urease solution. It was left to react for 2 hours with magnetic stirring at 4°C. Finally, 6 cycles of purification were carried out, where the urease was purified with Amicon® Pro Centrifugal Filters at 3600 rpm, for 30 minutes at 4°C.

The anti-human ferritin heavy chain 1 (FTH1) monoclonal antibody solution (in BBS pH 9) was dissolved in phosphate pH 7.4 solution. NHS-LL-Biotin solution was added to the antibody solution to achieve a concentration ratio of Antibody:NHS-LL-Biotin 1:25. The mixture was left to react for 2 hours with magnetic stirring at 4°C. Finally, 6 cycles of purification were carried out, where the antibody was purified with Amicon® Pro Centrifugal Filters at 3600 rpm, for 30 minutes at 4°C.

S3. Selection of measurement buffer

As said in the article, the nature of the measurement buffer was selected by analyzing the response of b-urease/Strept-gFETs after urea addition to different buffers (Figure S2).

The buffer with the best response was selected considering the slopes of the dependence of the I_{DS} as a function of time after urea addition. The slopes of the plots in **Figure S2** were 0.066 ± 0.0001 μA/s with a ΔI_{DS} of 25 μA for the buffer 0.1 mM sodium acetate (AcNa) in 100 mM NaCl (pH 7); 0.145 ± 0.0004 μA/s with a ΔI_{DS} of 40 μA for 0.01X PBS (pH=7.4), and 0.151 ± 0.0002 μA/s with a ΔI_{DS} of 47 μA for 0.1mM Hepes in 10mM KCl (pH=6). According to these results, the last buffer was selected for ELISA type measurements.

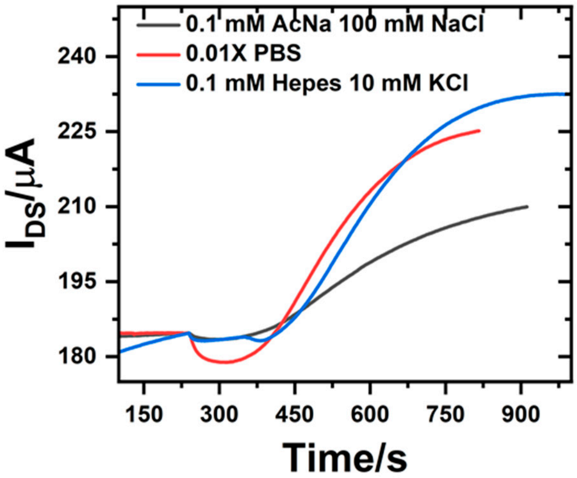

Figure S2. Changes in IDS after addition of 1 mM urea for a Strept-gFET incubated with different buffers (VDS = 50 mV, VGS = −250 mV).

S4. Antifouling strategy to improve specificity

Several antifouling strategies were carried out to decrease non-specific binding in the present study. Firstly, PEG-NH_2 was used after the functionalization step with the primary mAb-ferritin to integrate antifouling PEG units on the graphene surface. Second, the biointerface was blocked with BSA. Third, BSA and Tween 20 were added to the blocking buffer, and the buffer was used to prepare ferritin solutions. These antifouling strategies and the procedure until the first control can be seen in Figure S3A. In addition, a study with two G-ELISA sensors blocked with BSA and two sensors without the BSA blocking (Figure S3B) was performed to evaluate the BSA blocking effect. Figure S3C shows the comparison of performances between the sensors with (main Ms.) and without BSA blocking step. In the G-ELISA measurements without BSA blocking, a rapid saturation in the IDS curves and an increase in the dispersion of the slopes of the different ferritin concentrations can be observed when compared to two G-ELISAs that did have BSA blocking. Thus, although the measurements without BSA blocking show a lower response in terms of the slope of current variation over time, the values for the sensors with the BSA blocking step exhibit lower dispersions, resulting in more accurate determinations.

Furthermore, a study comparing two washing buffers was also performed for the selection of the best washing buffer: PBS (Figure S3D) and PBST (Figure S3E). It can be observed that the PBST buffer generates a better separation of the IDS curves for the different concentrations of ferritin compared with the I_{DS} curves obtained with the PBS washing buffer. A better linearity was also observed in the semilog plot of the slopes as a function of ferritin concentration (Figure S3F). These results indicate that the PBST buffer is a better washing buffer than the PBS buffer since it manages to reduce nonspecific binding achieving better resolution and greater reproducibility in the measurements.

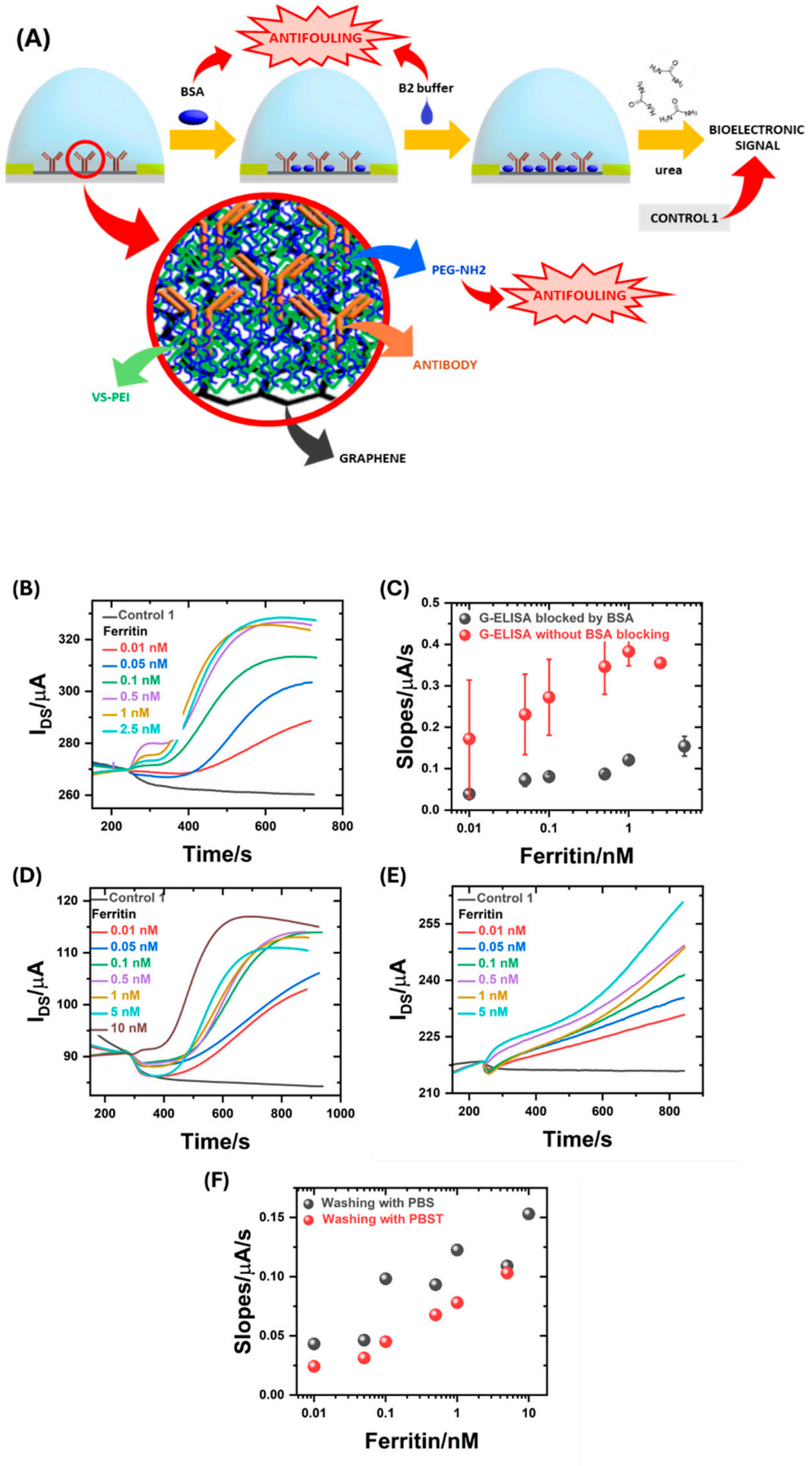

Figure S3. (A) G-ELISA scheme with antifouling strategies and measurement of control 1. (B) Changes in I_{DS} for a G-ELISA after incubation with different concentrations of ferritin and the addition of 1 mM urea, without the BSA blocking step (VDS = 50 mV, VGS = −250 mV, 0.1 mM HEPES buffer with 10 mM KCl, pH 6). (C) Comparison of G-ELISA slopes for two biosensors, one with the BSA blocking step and one without BSA blocking. (D) Changes in I_{DS} for a G-ELISA after incubation with different concentrations of ferritin and the addition of 1 mM urea, where washing steps were performed with PBS buffer pH 7.4 (V_{DS} = 50 mV, V_{GS} = −250 mV, 0.1 mM HEPES buffer with 10 mM KCl, pH 6). (E) Changes in the I_{DS} for a G-ELISA after incubation with different concentrations of ferritin and the addition of 1 mM urea, where washing steps were performed with PBST pH 7.4 buffer (V_{DS} = 50 mV, V_{GS} = −250 mV, 0.1 mM HEPES buffer with 10 mM KCl, pH 6). (F) Comparison

of the slopes of G-ELISA for two biosensors, with different washing buffers, one with PBS pH 7.4 and the other with PBST pH 7.4.

S5. G-ELISA measurement protocol:

Detailed protocol of the steps to follow to perform G-ELISA measurements. This protocol is presented the table below:

Table S1. Detailed protocol for G-ELISA measurements.

**1.**	Block for 20 minutes with 180 µl of 0.1% BSA								
**2.**	Wash 3 times in a row with 300 µl of PBS + 0.05% Tween 20								
**3.**	Block 10 minutes with 30 µl of 100 mM Hepes + 0.05% Tween 20 + 0.1% BSA								
**4.**	Wash 3 times in a row with 300 µl of PBS + 0.05% Tween 20								
**5.**	Wash 5 times in a row with 300 µl of 0.1 mM Hepes 10 mM KCl								
**6.**	Add 300 µl of 0.1 mM Hepes 10 mM KCl, measure the current for 4 minutes and add 3 µl of urea, measure the current another 10 minutes (Control 1)								
**7.**	Wash 3 times in a row with 300 µl of PBS + 0.05% Tween 20								
		**Control 2**	**Ferritin (nM)**						
**8.**	Incubate 50 µl of the different ferritin solutions for 15 minutes:	**PBS + 0.05% Tween 20 + 0.5% BSA**	**0.01**	**0.05**	**0.1**	**0.5**	**1**	**5**	**10**
**9.**	Wash 3 times in a row with 300 µl of PBS + 0.05% Tween 20								
**10.**	Incubate with 30 µl of b-mAb-Ferritin for 10 minutes								
**11.**	Wash 3 times in a row with 300 µl of PBS + 0.05% Tween 20								
**12.**	Incubate with 30 µl of streptavidin for 5 minutes								
**13.**	Wash 3 times in a row with 300 µl of PBS + 0.05% Tween 20								
**14.**	Incubate with 30 µl of b-urease for 5 minutes								
**15.**	Wash 3 times in a row with 300 µl of PBS + 0.05% Tween 20								
**16.**	Wash 5 times in a row with 300 µl of 0.1 mM Hepes 10 mM KCl								
**17.**	Add 300 µl of 0.1 mM Hepes 10 mM KCl, measure the current for 4 minutes and add 3 µl of urea, measure the current another 10 minutes								

S6. Interference study

An interference study was performed using concanavalin A (ConA) and glucose oxidase (GOx) as model proteins, both at a concentration of 10 nM (Figure S4). This study was carried out by comparing the % IDS response of these interfering proteins and ferritin. It can be seen that despite having a response with both interferential agents, they do not exceed the response obtained with 10 nM of ferritin. Furthermore, this nonspecific response may be due to nonspecific binding of biotinylated urease.

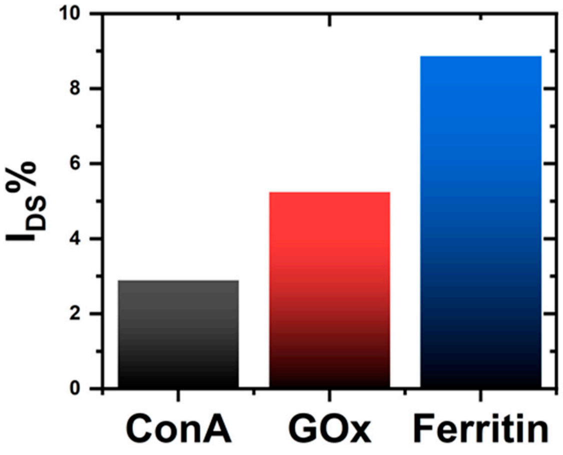

Figure S4. I_{DS}% at 900 seconds of G-ELISA with a concentration of 10 nM of ferritin, ConA and GOx.

S7. Stability study of mAb-ferritin-gFET

The stability of two mAb-ferritin-gFETs was studied over time. For this, I_{DS} *vs*. time G-ELISA measurements were performed at increasing ferritin concentrations one day after (day 1, Fig. S5A) the gFETs functionalization with mAb-ferritin. The mAb-ferritin-gFETs were recovered with a 10 mM glycine solution pH 2. After twelve days (day 12), biosensing measurements were carried out (Fig. S5B) and the responses measured each day were compared. Figure S5C shows the G-ELISA slope obtained at 5 nM ferritin at day 1 and 12. The results evidence good regeneration of the mAb-ferritin-gFETs and satisfactory stability of the G-ELISA activity over 12 days.

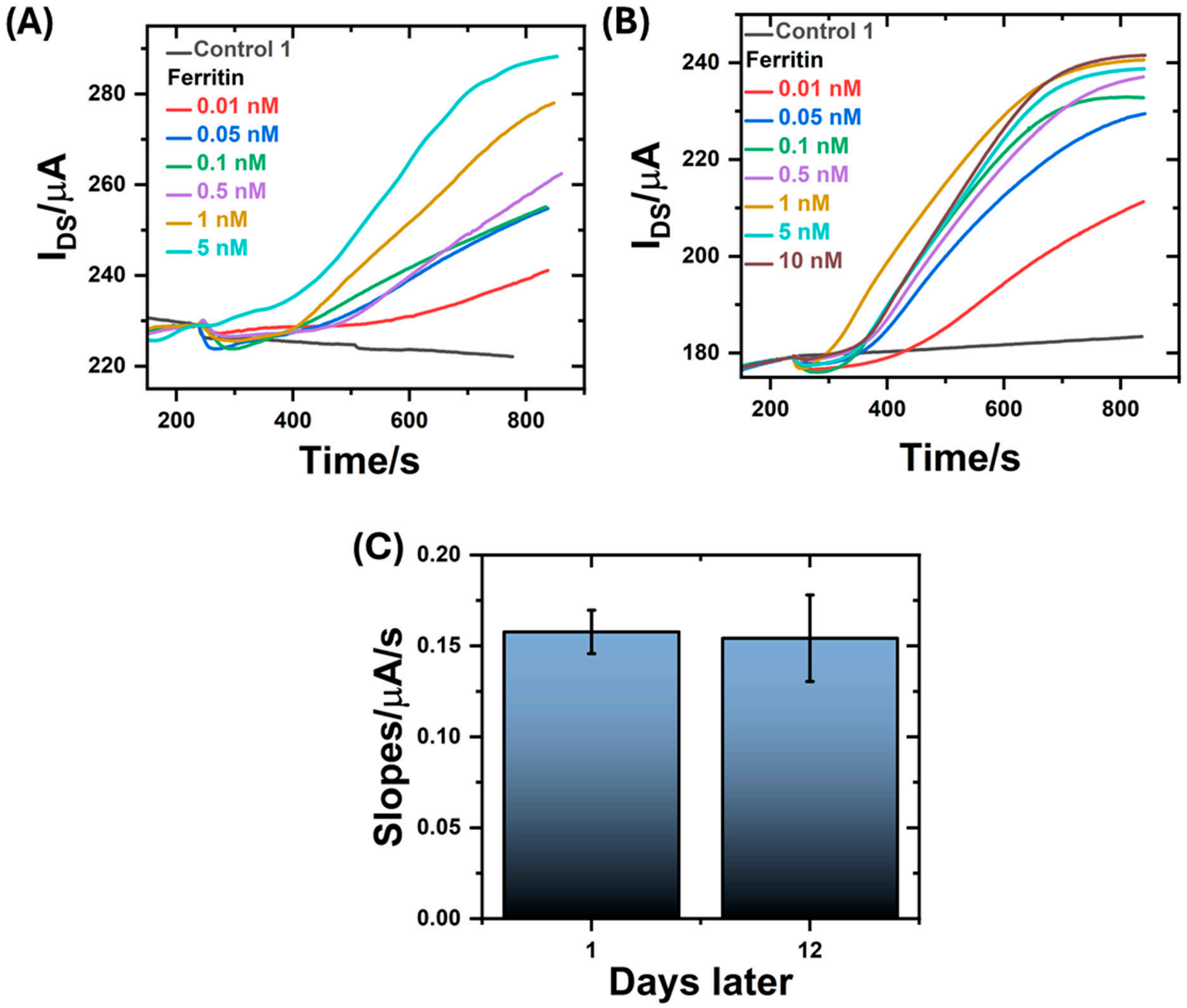

Figure S5. (A) Changes in IDS for a G-ELISA after incubation with different concentrations of ferritin and the addition of 1 mM urea (VDS = 50 mV, VGS = −250 mV, 0.1 mM HEPES buffer with 10 mM KCl, pH 6). (B) Changes in IDS for a G-ELISA after incubation with different concentrations of ferritin and the addition of 1 mM urea (VDS = 50 mV, VGS = −250 mV, 0.1 mM HEPES buffer with 10 mM KCl, pH 6) for one biosensor recovered and measured 12 days later. (C) Comparison of G-ELISA slopes with 5 nM ferritin for two biosensors recovered and measured 12 days later.

S8. non-specific binding study

A study was performed to identify at which incubation step we non-specific binding is more important. To do this, a sensor was first incubated with b-urease alone, washed and the enzymatic response was measured. Secondly, it was first incubated with streptavidin and then with b-urease and the second measurement of the enzymatic response was performed. And finally, it was incubated with b-mAb-Ferritin (the secondary antibody), streptavidin and b-urease, and the current response to the enzymatic reaction was measured. As can be seen in Figure S6A, there is a non-specific binding by b-urease and this non-specific binding increases in the incubation with streptavidin. This may be due to the biotinylation step of urease, where we may have bound too much concentration of biotin to urease and generated this non-specific binding. Furthermore, it can be seen in Figure S6B that 64% of the I_{DS}% of control 2 is due to the b-urease response, while in the streptavidin I_{DS}% only 42% can be attributed to non-specific binding of b-urease, so streptavidin is contributing a significant part of the current increase due to non-specific binding.

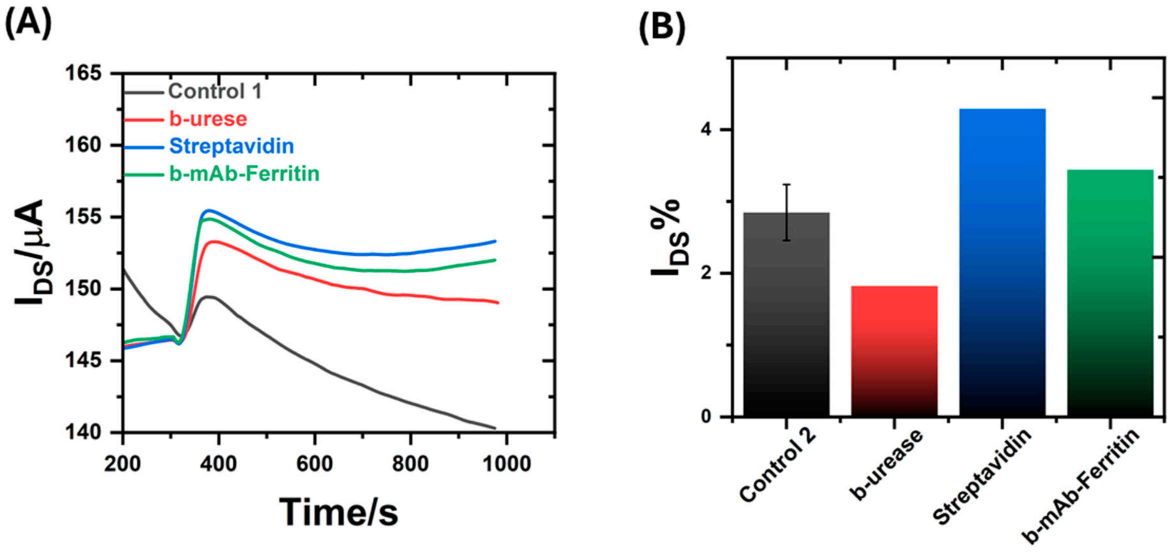

Figure S6. (A) Changes in I_{DS} for a mAb-Ferritin-gFET after incubation with b-urease, incubation with streptavidin and b-urease, and finally incubation with b-mAb-Ferritin, streptavidin, and b-urease and addition of 1 mM urea (V_{DS} = 50 mV, V_{GS} = −250 mV, 0.1 mM HEPES buffer with 10 mM KCl, pH 6).(B) Comparison of IDS% at 900 seconds of control 2 versus nonspecific binding of b-urease, streptavidin-b-urease, and b-mAb-ferritin-streptavidin-b-urease.

S9. Demonstration of the portability and ease of use of the reading device.

For this purpose, a video was made showing the portability of the portable FET measuring station Zaphyrus-W10 and its ease of use as a reading device.

Link to the video: https://youtu.be/Gpv7iBX3q88?si=pUNkR-k3aCAgyV6V (accessed on 15 August 2024)
